# A Recent HIV Diagnosis Is Associated with Non-Completion of Isoniazid Preventive Therapy in an HIV-Infected Cohort in Cape Town

**DOI:** 10.1371/journal.pone.0052489

**Published:** 2012-12-20

**Authors:** Tolu Oni, Relebohile Tsekela, Bekekile Kwaza, Lulama Manjezi, Nonzwakazi Bangani, Katalin A. Wilkinson, David Coetzee, Robert J. Wilkinson

**Affiliations:** 1 Clinical Infectious Diseases Research Initiative, Institute of Infectious Diseases and Molecular Medicine, Faculty of Health Sciences, University of Cape Town, Cape Town, South Africa; 2 Division of Medicine, Imperial College London, London, United Kingdom; 3 Centre for Infectious Disease and Epidemiology Research, School of Public Health, University of Cape Town, Cape Town, South Africa; 4 Khayelitsha Day Hospital, Site B Khayelitsha, Cape Town, South Africa; 5 Medical Research Council, National Institute for Medical Research, London, United Kingdom; San Francisco General Hospital, University of California San Francisco, United States of America

## Abstract

**Introduction:**

Despite high rates of successful treatment TB incidence in South Africa remains high, suggesting ongoing transmission and a large reservoir of latently infected persons. Isoniazid preventive therapy (IPT) is recommended as preventive therapy in HIV-infected persons. However, implementation has been slow, impeded by barriers and challenges including the fear of non-adherence.

**Objective and Methods:**

The aim was to evaluate predictors of IPT non-completion. One hundred and sixty four antiretroviral therapy (ART)-naïve HIV-infected patients with tuberculin skin test ≥5 mm were recruited from Khayelitsha day hospital and followed up monthly. A questionnaire was used to collect demographic information.

**Results:**

The overall completion rate was 69%. In multivariable analysis, there was a 29% decrease in risk of non-completion for every year after HIV diagnosis (OR 0.81; 95% C.I. 0.68–0.98). Self-reported alcohol drinkers (OR 4.05; 95% C.I. 1.89–9.06) also had a four-fold higher risk of non-completion, with a strong association between alcohol drinkers and smoking (χ^2^ 27.08; p<0.001).

**Conclusion:**

We identify patients with a recent HIV diagnosis, in addition to self-reported drinkers and smokers as being at higher risk of non-completion of IPT. The period of time since HIV diagnosis should therefore be taken into account when initiating IPT. Our results also suggest that smokers and alcohol drinkers should be identified and targeted for adherence interventions when implementing IPT on a wider scale.

## Introduction

Tuberculosis (TB) represents a threat to global public health, despite the availability of efficacious treatment, with a third of people estimated to be latently infected worldwide, 33.2 million with HIV infection [1]. HIV-1 (HIV) is the strongest known risk factor for TB and where these pandemics intersect, they are the most common cause of death among young adults in many countries. Sub-Saharan Africa bears the brunt of this burden with the World Health Organization (WHO) Africa region having the highest estimated incidence rate [2].

In response to these dual epidemics, the WHO’s STOP TB strategy to reduce the global burden of TB by 2015, recommends collaborative HIV/TB activities including the three “I”s for HIV/TB: Intensified Case-Finding (ICF), Isoniazid Preventive Therapy (IPT), and Infection Control in addition to antiretroviral therapy (ART) [3]. IPT has been shown to significantly reduce the risk of incident TB [4] predominantly in those with positive tuberculin skin test. This benefit depends on good adherence to the treatment regime. While ART roll-out has been expanding, implementation of IPT has been slow, impeded by barriers and challenges. This lack of implementation is in part due to fear of non-adherence [5].

South Africa is a high TB burden setting with an estimated incidence rate in 2010 of 981/100,000, and with 60% of TB cases occurring in HIV-infected persons [6]. This makes IPT crucial to TB control in this high-burden setting. IPT is slowly being implemented in the public health sector. WHO estimates of the number of people on IPT in South Africa show an increase from 7359 in 2008 to 124,049 persons in 2010 [6], [7]. However, this represents a small fraction of people eligible to receive IPT. Furthermore, this does not elaborate on the proportion of persons initiating IPT that complete the course. Literature on adherence to IPT from higher-income settings have shown varying IPT completion rates from 45% in the USA [8] to 81% in Spain [9]. Factors identified to be associated with non-adherence, including younger populations, males, smokers and recent migrants, have also been shown to vary across different population groups [8]–[12]. This highlights a need for local data on completion rates and predictors of non-completion of IPT amongst eligible persons.

We report on completion rates of IPT within a research context and patient-related predictors of non-completion as a means of identifying those at high risk of non-adherence.

## Methods

### Study Design, Setting and Population

Participants in this study were identified from a larger cross-sectional TB diagnostics study to develop biomarkers that distinguish active from latent TB and other infections. The study was conducted at site B Day Hospital, a primary care health facility in the informal township of Khayelitsha. Located 30 km from Cape Town, Khayelitsha has a population of over 400,000 and a TB case notification rate of over 1600/100 000, 70% of which occur in HIV co-infected persons [13].

### Ethics Statement

Written informed consent was obtained from all participants recruited as part of a larger TB diagnostics study. This study was approved by the University of Cape Town health research ethics committee (REC 012/2007).

### Sampling and Recruitment Strategy

Study participants were recruited from the HIV wellness clinic, comprising patients not eligible for antiretroviral therapy (ART) at Khayelitsha day hospital, between February 2008 and March 2010. Participants were ART-naive with CD4≥200 cells/mm^3^, either newly diagnosed or attending for minor ailments. Consecutive patients attending the clinic without any symptoms of TB were invited to participate, tested for evidence of latent TB and screened for active TB using a symptom screen, sputum microscopy and culture and chest radiography. Latent TB (LTBI) was diagnosed in these HIV-infected persons by the tuberculin skin test (TST) using 2 TU of tuberculin PPD RT23 injected intradermally into the volar aspect of the forearm and read 48–72 hours later by a dedicated study nurse. A transverse induration diameter ≥5 mm was considered as evidence of LTBI. Participants were considered alcohol drinkers if they reported regular alcohol consumption. Smokers were defined as participants who self reported current smoking and non-smokers as never- or ex-smokers.

Asymptomatic persons identified with a skin induration diameter of ≥5 mm and no evidence of active TB disease were given information about the implications of infection with latent TB infection and the risk of progression to active TB, and commenced on IPT for six months, as per South African national guidelines. Participants were seen at monthly visits to monitor adherence and side effects, and the benefit of IPT in HIV-infected persons and importance of completion of the course reinforced by the study investigator at every visit. Adherence was measured by self-reported adherence on attendance of follow-up appointments. The same investigator ascertained self-reported adherence at these visits by asking patients if they took the pills as prescribed everyday in the preceding month. Patients who missed an appointment received a daily telephonic reminder and failing that after a week, a home visit by the trained clinical research assistant to encourage clinic attendance. As IPT was dispensed at monthly follow-up visits, non-completion of IPT (outcome variable) was defined as a failure to attend all six monthly appointments within a nine-month period.

In addition, a questionnaire, applied by trained study personnel, was used to collect baseline demographic information including age, employment status, alcohol consumption, smoking status, past TB history, recent TB contact, type of accommodation lived in, and date of HIV diagnosis. This questionnaire was designed for the larger TB diagnostics study and data for this study extracted from the database. No study incentives were provided to participants. We hypothesized, based on existing literature, that male sex, being employed, consuming alcohol and smoking would be predictors of non-completion of IPT.

### Statistical Analysis

Patient baseline characteristics were stratified by outcome and summarised using simple proportions. Predictors of non-completion were analysed by logistic regression and nested models compared using the likelihood ratio test. The Chi squared test was used to compare proportions. The Akaike’s Information Criterion (AIC) was used to compare non-nested models with a lower AIC indicating a better model. The interactions between confounding and exposure variables were then examined. The fit of the model was assessed using Pearson’s goodness-of-fit test with a p-value >0.05 indicating a good fit. Model diagnostics were performed on final models by checking the form of the linear predictors. Significance testing was done using two-sided p-values and 95% confidence intervals. All data were analysed using STATA 10.0 (StataCorp, College Station, TX, USA).

## Results

### Baseline Characteristics and IPT Completion Rate

**Figure 1:**
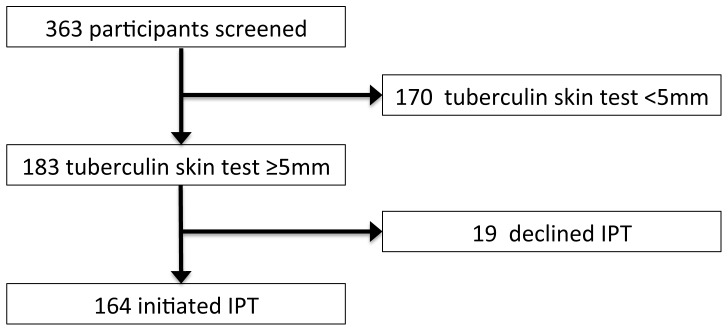
Flow diagram of screening and enrolment into study.

Of the three hundred and fifty three asymptomatic participants screened, one hundred and sixty four participants identified with LTBI were initiated on IPT (Figure 1). Overall, one hundred and thirteen persons (69%) completed the six-month IPT course within a nine-month period. Participants that attended all appointments and completed the six-month course reported taking dispensed pills from the preceding month as prescribed.

Baseline characteristics were compared, stratified by completion or non-completion of a six-month course of IPT, and summarized in Table 1. Amongst those that did not complete the IPT course, a median of two months IPT (IQR 0–3 months) was completed. Compared to those who completed IPT, patients who did not complete the course had a higher proportion of smokers (21.6% versus 9.7%; p = 0.04) and alcohol drinkers (37.2% versus 14.2%; p = 0.001). They were more likely to have been recently diagnosed with HIV (median time since HIV diagnosis 54 days (IQR 6–910) versus 421 days (IQR 43–1479); p = 0.002). There were also a higher proportion of men versus women (22.4% versus 15%; p = 0.252) in the non-completers group, although not statistically significant. Similarly, a greater proportion of non-completers were recent migrants to Khayelitsha (11.9% versus 5.3%; p = 0.155) although numbers were small. The distribution of all other measured baseline characteristics were similar between the two groups.

**Table 1:** Baseline characteristics of HIV-infected persons initiating isoniazid preventive therapy who did and did not complete 6 months of IPT.

Characteristics		Completers N = 113N (%)	Non-completers N = 51N (%)	Univariable analysis OR (95% C.I.)
Gender	Female	96 (85.0)	39 (76.5)	2.34 (0.97–5.64)
Marital status	Married	33 (29.2)	10 (19.6)	0.93 (0.40–2.14)
Smoker	Yes	11 (9.7)	11 (21.6)	3.59 (1.41–9.18)
Alcohol	Yes	16 (14.2)	19 (37.2)	4.66 (2.03–10.69)
TB contact	No Yes Don’t know	78 (69.0) 30 (26.6) 5 (4.4)	41 (80.4) 10 (19.6) 0 (0.0)	0.54 (0.23–1.26)
Previous TB	Yes	15 (13.3)	6 (11.8)	0.94 (0.32–2.79)
BCG Scar	Yes	62 (54.9)	23 (45.1)	0.92 (0.44–1.94)
Self–reported BCG	No Yes Don’t know	19 (16.8) 80 (70.8) 14 (12.4)	11 (21.6) 33 (64.7) 7 (13.7)	1.13 (0.58–2.22)
Employed	Yes	41 (36.6)	17 (33.3)	0.85 (0.39–1.84)
Accommodation	Shack House	66 (58.4) 47 (41.6)	21 (50.0) 21 (50.0)	1.46 (0.69–3.06)
Time in Khayelitsha	<1 year > = 1 year	6 (5.3) 107 (94.7)	5 (11.9) 37 (88.1)	0.39 (0.11–1.36)
Characteristics		Median (IQR)	Median (IQR)	OR (95% C.I.)
Age (years)		32.7 (27.4–37.8)	29.8 (24.5–35.9)	0.99 (0.94–1.04)
BMI		27.2 (22.8–31.6)	24.2 (21.3–30.5)	0.96 (0.90–1.02)
Education	Highest school grade achieved	11 (9–12)	11 (10–12)	1.07 (0.90–1.29)
Persons/bedroom		2.33 (1.67–3)	2 (2–3)	0.91 (0.68–1.20)
CD4 count (cells/mm3)		360 (269–508)	363 (261–526)	1.00 (1.00–1.00)
Years since HIV diagnosis		1.15 (0.12–4.05)	0.15 (0.02–2.49)	0.82 (0.67–0.99)

### Predictors of Non-completion of IPT

On univariable analysis, being male, smoking, alcohol, and a recent HIV diagnosis were associated with an increased risk of IPT non-completion (Table 1). There was a significant association between being male and drinking alcohol (p = 0.002) and smoking (p<0.001). There was also a strong association between smoking and drinking (p<0.001).

On multivariable analysis the final logistic regression model included alcohol and time since HIV diagnosis variables only. There was a 19% decrease in odds of non-completion with every year after HIV diagnosis (OR 0.81; 95% C.I. 0.68–0.98; p = 0.03) and a four-fold increase in odds of non-completion in drinkers compared to non-drinkers (OR 4.05; 95% C.I. 1.89–9.06; p = 0.001).

Due to the strong association between smoking and drinking we chose to include the alcohol variable, as its inclusion in the model resulted in a marginally better fit of the model to the data using the Pearson goodness of fit test compared to a model that included smoking. However, given the association between these two variables, both should be considered in the interpretation of these results. There was no significant effect modification between gender and drinking or smoking although it should be noted that a greater proportion of male versus female participants reported being smokers (46% versus 7%) and drinkers (43% versus 16%).

**Figure 2:**
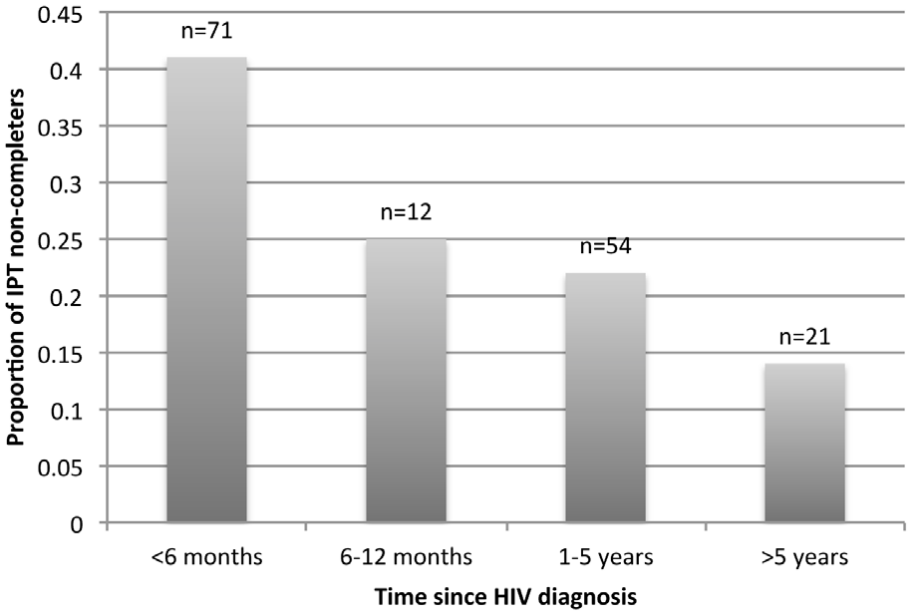
Histogram showing the proportion of IPT non-completers stratified by time period since HIV diagnosis. N represents the total sample size of participants in each time period.

We further explored the relationship between the period of time since HIV diagnosis and non-completion of IPT by examining the proportion of non-completers stratified into four time periods since HIV diagnosis (<6 months, 6–12 months, 1–5 years, and >5 years since HIV diagnosis). Figure 2 shows that non-completers were most likely to default IPT if initiated within six months of HIV diagnosis compared to persons initiated after at least six months post-HIV diagnosis (p = 0.006).

## Discussion

We report a 69% IPT completion rate in this ART-naïve HIV-infected population in Cape Town suggesting that adherence in this setting is a potential major challenge to achieving full benefit from this public health intervention. Furthermore we found that non-completion of IPT was associated with a recent HIV diagnosis and alcohol use. Studies conducted in similar high burden settings have reported IPT completion rates from 47% in HIV-infected adults attending clinics in Limpopo, rural South Africa to 78% in participants involved in a randomized controlled trial in Botswana [14], [15].

These results highlight a need for innovative strategies to improve adherence and recent studies have explored interventions using cellular telecommunication networks and mobile phones [16].

Studies examining risk factors for non-completion have reported male patients as being at risk of non-completion [15], [17] with men being twice as likely to be non-completers and women being three times as likely as men to complete the course, respectively. We explored quantitative patient predictors of non-completion of IPT and our results were coherent with existing literature [11], [18], with alcohol drinking and smoking found to significantly increase the risk of non-completion. In this high-burden setting, this association possibly relates to marginalization representing a social risk factor. This was demonstrated by a study in Spain that reported an association between non-adherence and patients with unfavourable social circumstances such as alcohol and drug abuse and recent immigrants [9].

In addition, we also found patients with a more recent HIV diagnosis had a higher risk of non-completion. This could relate to non-acceptance of HIV status and a resulting reluctance to attend follow-up appointments. It could also relate to non-disclosure of HIV status to friends and family and wanting to keep their HIV status confidential, resulting in an unwillingness to attend the health facility. In addition, this finding could be related to their perceived risk and lack of education on HIV opportunistic infections such as TB, information often received at HIV clinics. A study conducted in Thailand reported acceptance of personal HIV status as a predictor of adherence [17]. Patients established in HIV clinics are potentially more likely to have accepted their HIV status and therefore more likely to have developed a habit of attending the health facility for routine appointments and for minor ailments, which could facilitate IPT adherence. However, barriers such as transport costs to the health facility and a reluctance to take medication when feeling healthy could impact adherence even among patients established in care. Our study did not assess reasons for the association and these potential reasons for non-completion of therapy should be investigated in further study. Based on these findings, it is possible that provision of comprehensive counseling to patients with a recent HIV diagnosis, as offered prior to ART initiation, may lead to better acceptance of HIV status and good adherence to IPT.

Studies in high-income settings have demonstrated that provision of shorter LTBI regimens under research [19] is associated with better adherence [20]. Therefore it is possible that new shorter regimens could significantly improve adherence and completion rates.

Based on existing literature, we hypothesized that male and unemployed patients would also be at higher risk of non-completion. Unemployment rates are high in Khayelitsha and this could explain why employment was not associated with adherence in our study. Sixty-five percent of drinkers and 59% of smokers were male suggesting that the expected association between male sex and non-completion of IPT could relate to a greater proportion of alcohol drinkers and smokers within this group and could explain why being male was not a significant risk factor in this study.

### Limitations

Although IPT follow-up was conducted within the health facility, it was conducted within a research context and it is likely that adherence in operational settings will be lower than in a research study. Our measurement of completion was IPT was based on self-reported adherence, which may overestimate the proportion of completers. However, all participants that reported good adherence attended all six monthly visits, suggesting good self-motivation and making it more likely that they completed the IPT course. Substance abuse, commonly reported in this community and a possible risk factor for non-completion to be considered in the wider implementation of IPT, was not measured in this study. The quantity and frequency of alcohol use was not recorded and it is possible that the association between non-adherence and alcohol use is restricted to excessive or binge drinking. Healthcare worker related barriers to IPT implementation were identified by Getahun *et al*, with lack of experience and knowledge of current guidelines cited as an important barrier [7]. Another barrier to implementation identified includes fear of side effects with hepatitis and peripheral neuropathy most common [17], [21]. Our study did not examine the impact of healthcare worker related barriers or IPT side effects on adherence.

### Conclusions

The WHO has identified IPT as one of the main interventions to reduce morbidity and mortality from TB in people living with HIV. A better understanding of predictors on non-adherence to IPT is therefore crucial to optimize adherence in high HIV/TB burden settings. Implementation of IPT has been slow in South Africa. The findings of this study are therefore important as the completion rate reported demonstrates the potential feasibility of providing IPT in this setting.

Retention of pre-ART patients in care is often challenging. Provision of IPT, as part of a package of care including counseling and cotrimoxazole prophylaxis, could therefore provide a means of improving retention in care, especially for patients recently diagnosed with HIV.

The identification of a recent HIV diagnosis as a significant predictor of non-completion suggests that in this setting, better strategies and interventions will be required to improve adherence to IPT in recently diagnosed HIV-infected persons. Our results also suggest that when initiating IPT in this setting, the smoking and alcohol history of patients should be ascertained, with targeted adherence interventions implemented aimed at those who smoke or drink in addition those with a more recent HIV diagnosis.
